# Maternal exposure to air pollution alters energy balance transiently according to gender and changes gut microbiota

**DOI:** 10.3389/fendo.2023.1069243

**Published:** 2023-04-04

**Authors:** Olivia Pizetta Zordão, Clara Machado Campolim, Victor Yuji Yariwake, Gisele Castro, Clílton Kraüss de Oliveira Ferreira, Andrey Santos, Sónia Norberto, Mariana Matera Veras, Mario Jose Abdalla Saad, Paulo Hilário Nascimento Saldiva, Young-Bum Kim, Patricia Oliveira Prada

**Affiliations:** ^1^ Department of Internal Medicine, School of Medical Science, State University of Campinas (UNICAMP), Campinas, SP, Brazil; ^2^ Laboratory of Environmental and Experimental Pathology, Department of Pathology, University of Sao Paulo School of Medicine, Sao Paulo, SP, Brazil; ^3^ School of Applied Sciences, State University of Campinas (UNICAMP), Limeira, SP, Brazil; ^4^ Division of Endocrinology, Diabetes and Metabolism, Department of Medicine, Beth Israel Deaconess Medical Center and Harvard Medical School, Boston, MA, United States

**Keywords:** obesity, air pollution, metabolic program, particulate matter, gut microbiota, PM2.5, inflammation, gestation

## Abstract

**Introduction:**

The timing of maternal exposure to air pollution is crucial to define metabolic changes in the offspring. Here we aimed to determine the most critical period of maternal exposure to particulate matter (PM_2.5_) that impairs offspring's energy metabolism and gut microbiota composition.

**Methods:**

Unexposed female and male C57BL/6J mice were mated. PM_2.5_ or filtered air (FA) exposure occurred only in gestation (PM_2.5_/FA) or lactation (FA/PM_2.5_). We studied the offspring of both genders.

**Results:**

PM_2.5_ exposure during gestation increased body weight (BW) at birth and from weaning to young in male adulthood. Leptin levels, food intake, Agrp, and Npy levels in the hypothalamus were also increased in young male offspring. Ikbke, Tnf increased in male PM_2.5_/FA. Males from FA/PM_2.5_ group were protected from these phenotypes showing higher O_2_ consumption and Ucp1 in the brown adipose tissue. In female offspring, we did not see changes in BW at weaning. However, adult females from PM_2.5_/FA displayed higher BW and leptin levels, despite increased energy expenditure and thermogenesis. This group showed a slight increase in food intake. In female offspring from FA/PM_2.5_, BW, and leptin levels were elevated. This group displayed higher energy expenditure and a mild increase in food intake. To determine if maternal exposure to PM_2.5_ could affect the offspring’s gut microbiota, we analyzed alpha diversity by Shannon and Simpson indexes and beta diversity by the Linear Discriminant Analysis (LDA) in offspring at 30 weeks. Unlike males, exposure during gestation led to higher adiposity and leptin maintenance in female offspring at this age. Gestation exposure was associated with decreased alpha diversity in the gut microbiota in both genders.

**Discussion:**

Our data support that exposure to air pollution during gestation is more harmful to metabolism than exposure during lactation. Male offspring had an unfavorable metabolic phenotype at a young age. However, at an older age, only females kept more adiposity. Ultimately, our data highlight the importance of controlling air pollution, especially during gestation.

## 1 Introduction

Obesity has become a global health challenge reaching alarming prevalence and being a cause of chronic metabolic diseases, including type 2 diabetes (T2DM) (1–3). The etiology of obesity implicates multiple factors involving environmental, genetic, and epigenetic modification (4–7). Clinical, epidemiological, and experimental studies consistently demonstrate that abnormal maternal caloric intake or environment disturbance during the perinatal period favors the development of cardiometabolic diseases in the offspring (6, 8).

The mechanism by which an altered environment during the perinatal period induces obesity remains unknown (8). The hypothalamus is the most studied organ that integrates peripheral signals with neural circuits and nutritional signals, keeping food intake and energy expenditure (EE) in balance (9–11). The arcuate nucleus of the hypothalamus (ARC) has a diversity of neuron populations that control metabolism. Proopiomelanocortin (Pomc) and Agouti-related peptide (Agrp) co-located with neuropeptide Y (Npy) are the most studied that control energy balance. Fasting activates the orexigenic Agrp/Npy neurons inducing food intake (9–14).

In contrast, feeding activates Pomc neurons, suppressing feeding while increasing energy expenditure (15). Failure to develop Pomc and Agrp neurocircuits increases the predisposition to obesity and its comorbidities (8, 16–18). Changes in the maternal diet composition containing a significant amount of saturated fat cause obesity and glucose intolerance (19) and elicit a preference for sucrose (15). Factors other than excess nutrients consumed by the mother can alter the metabolic phenotype of her offspring. Maternal smoking, even at a low-intensity frequency during gestation, is associated with reduced fetal growth and an increased risk of preterm birth (20).

Air pollution exposure containing particulate matter with a small diameter (≤ of 2.5 μm: PM_2.5_) increases the risk of obesity and T2DM (21–23). The ability of PM_2.5_ to induce metabolic diseases is based mainly on inflammatory responses induced by its chemical composition, which can also include lipopolysaccharides (LPS) (24–28). LPS is a potent agonist of Toll-Like Receptor 4 (TLR4), considered a part of the etiopathology of obesity and T2DM (25, 29). TLR4 utilizes a canonical signaling cascade activating the upstream nuclear factor of kappa light polypeptide gene enhancer in B-cells inhibitor, alpha (IκB), mainly by inhibitor-IκB kinase (IKK)s, which phosphorylate the IκB, inducing its degradation and releasing the Nuclear Factor Kappa B (NFκB), a transcription factor, to migrate to the cellular nucleus. NFκB in the nucleus increases the transcription of proinflammatory genes such as Tumor Necrosis Factor-alpha (Tnf alfa) (30, 31) and the expression of IKKϵ (IKK epsilon) and TANK-binding kinase 1(TBK1) (32), perpetuating the inflammatory signal. In parallel to inflammation, IKKβ (IKK beta) and IKKϵ are kinases involved in developing insulin resistance and T2DM (33–35). Therefore, the activation of TLR4/NFκB and the IKKϵ pathways are key for inflammatory responses and metabolic dysregulation in numerous tissues.

Recently we showed that short-term exposure (five days) to PM2.5 was sufficient to increase the expression of inflammatory markers such as Tlr4, Ikbke, and Tnf alpha in the hypothalamus causing leptin resistance and obesity at long-term of PM2.5-exposure (36). Other groups have shown that PM exposure activates the NFκB pathway *in vivo* (mice) and culture cells from different tissues (37, 38).

Due to its lower diameter, PM_2.5_ can spread into tissues through the lungs and olfactory epithelium (27). A portion of PM_2.5_ can also be transported to the oropharynx and ingested, reaching the gastrointestinal tract and altering gut permeability and local microbiota (31, 32). Changes in the gut morphophysiology and microbiota facilitate inflammatory responses and might be related to causing metabolic diseases such as obesity and T2DM (39–41).

Evidence suggests that perinatal exposure to PM_2.5_ might alter the energy metabolism of the offspring (42–50). However, the results are divergent. It is challenging to compare these studies due to the different approaches and protocols for PM_2.5_ exposures. For instance, some studies employed intratracheal instillation of liquid diesel exhaust PM_2.5_ (48, 51). In contrast, other studies used natural PM_2.5_ or filtered air (FA) captured by ambient chambers (49, 50, 52). Besides, the timing points elected to maternal exposures are considerably distinctive among studies, including preconception or offspring exposures in some of them (48–50), which leads to maternal inflammatory responses before conception and direct contact of animals with the particles.

Considering that the time point of maternal PM_2.5_ exposure is crucial to determine the impact on the metabolic outcomes, specifically on the regulation of feeding and energy expenditure, and to tease apart the effect of preconception maternal exposure to PM_2.5_, in the present study, we exposed mothers to PM_2.5_ only during gestation or lactation. We had a control group that received FA simultaneously, and we kept the mothers and offspring on a chow diet. Using this paradigm, we investigated whether exposure to PM_2.5_ during gestation or lactation alters the metabolic phenotype throughout the offspring’s life. More specifically, we investigated possible changes in 1) body weight, fat mass and lean mass, leptin, food intake, and energy expenditure; 2) hypothalamic neuropeptides and pro-inflammatory markers; 3) the gut microbiota diversity and composition.

## 2 Materials and methods

### 2.1 Ethical committee approval

All experiments, animal handling, and breeding were performed following the National Institute of Health guidelines for experimental animals’ use and the approval of the Care of Animals and Ethical Committee for Animal Research of the State University of Campinas (CEUA Protocol 4627-1).

### 2.2 Animals and diet

The multidisciplinary center for biological research from the State University of Campinas (Sao Paulo, Brazil) provided eight-week-old male and female C57BL/6J mice (CEUA Protocol 4627-1). The animal facility displayed the following features: constant light/dark cycle (12 h/12 h), room temperature (22°C), humidity, and a high-efficiency particulate air filter (HEPA) receiving a standard rodent chow (3.39 kcal/g; Nuvilab CR-1, Nuvital Quimtia, Brazil) and water *ad libitum*.

After one week of acclimation, we set up breeding cages with two females and one male. Usually, three days were sufficient to initiate the gestation of at least one female. We randomly divided the females into three experimental groups on the first day of gestation:

1. Mothers exposed to PM_2.5_ during gestation and FA during lactation: PM_2.5_/FA (n=8)
2. Mothers exposed to FA during gestation and PM_2.5_ during lactation: FA/PM_2.5_ (n=8)
3. Mothers exposed to FA during gestation and lactation: FA/FA (n=8)

We recorded the birth weight of each puppy, and the number of puppies per mother was normalized (n=6-8). At weaning (21 days of age), female and male offspring were separated in plastic cages, receiving only a standard diet and water *ad libitum*. Food intake and body weight were measured weekly. The offspring received only FA from birth to euthanasia. We described all mice ages in the Results and Legends.

### 2.3 Exposure to PM_2.5_ or filtered air

The exposures were performed using the ambient particle concentrator (Harvard Ambient Particle Concentrator, HAPC) at the University of São Paulo (USP) in São Paulo, Brazil. We followed the same protocol from previous studies (39, 53–55). Briefly, the HAPC concentrates the ambient particles in the environment, separating fine particles *via* filters according to aerodynamic sizes, concentrating them from ambient air. The HAPC has two chambers, one filled with concentrated particulate matter (PM_2.5_) and another with additional filters releasing filtered air (FA). Female (mothers) mice were placed into the chambers according to the groups. Animals were exposed to 600 μg/m^3^ of PM_2.5_. The exposure concentration dose was defined based on the cumulative daily dose of exposure (24 hs) of a resident from the city of São Paulo. In São Paulo city, the current annual mean level of PM_2.5_ is around 25 μg/m³ in 24 hours (the concentration established by World Health Organization). We characterized the PM_2.5_ composition along the course of the experiments consisting of black carbons, polycyclic aromatic hydrocarbon, and metal trace elements such as Na, Al, Si, P, S, K, Ca, Ti, V, Fe, Ni, Cu, Zn, Pb (53–56). The exposure lasted about one hour and a half, and the mothers returned immediately to their home cages after each exposure.

### 2.4 Schematic framework

<inline-graphic mimetype="image" mime-subtype="tiff" xlink:href="fmars-09-1005534-g001.tif"/>

#### 2.4.1 Food intake determination

Mice at 13 weeks adapted for 24 h in individual metabolic cages. We recorded food intake for five consecutive days. We calculated the average daily food intake by subtracting the remaining food at the metabolic cage from the delivered food for each mouse.

#### 2.4.2 Energy expenditure

After 24 h of acclimation, we placed random fed male or female offspring in the Oxymax Deluxe System (CLAMS) (Columbus Instruments, Columbus, OH, USA) to measure oxygen (O_2_) consumption, carbon dioxide (CO_2_), and respiratory exchange ratio (RER). CLAMS was done in mice at 18-19 weeks.

#### 2.4.3 Hormones measurements

We collected serum from mice at 8-12 weeks old and 30 weeks old. For the young mice, blood was collected from the tail in overnight fasted mice. Blood was collected after euthanization (EU) by decapitation for the older mice. Decapitation was done in overnight fasted mice who received an overdose of a mixture of ketamine hydrochloride (300 mg/kg) and xylazine hydrochloride (30 mg/kg) *via* intraperitoneal (IP) injection and after the animal lost the reflexes. After blood collection, the serum was obtained *via* centrifugation and was stored at -80°C until hormone analysis. We determined serum leptin (#EZML-82K, Millipore; Billerica, MA, USA) levels with a commercial ELISA kit (36, 57–62).

### 2.5 Tissue collection for mRNA and gene expression by qPCR analysis

Mice (18-20 weeks) were euthanized by decapitation after an overdose of ketamine hydrochloride (300 mg/kg) and xylazine hydrochloride (30 mg/kg) *via* IP injection. BAT and the hypothalamus were dissected, frozen in N2, and stored at -80°C until RNA analysis. As previously described (58, 60), RNeasy Mini Kit from Qiagen (cat#74106; Qiagen Inc, CA, USA), Nanodrop 2000, High-Capacity cDNA Reverse Transcription Kit (cat#4368814, Applied Biosystems, CA, USA), TaqMan and QuantStudio 6 Flex Real-Time PCR System (#4485694) and Data Assist™ software Applied Biosystems, CA, USA) were used to get the relative expression levels of the genes. Used primers were: Npy, Mm003048253_m1; Agrp, Mm00475829_g1; Pomc, Mm00435874_m1; Ikbke, Mm00444862_m1; Ucp1, Mm01244861_m1; Tnf alpha, Mm00443258_m1; Tlr4, Mm00445273_m1; Hprt, Mm01545399_m1.

### 2.6 Microbiota analysis

Mice (30 weeks) were euthanized by decapitation after an overdose of ketamine hydrochloride (300 mg/kg) and xylazine hydrochloride (30 mg/kg) *via* IP injection. We collected fecal samples (liquid nitrogen) from mice and stored them at –80°C until we used them. We collected genomic DNA from samples using a QIAamp DNA Stool Mini kit (Qiagen, Hilden, Germany). We amplified the V3–V4 hyper-variable region of the bacterial 16S rRNA gene.

Next, we use the Illumina 16S Metagenomic Sequencing Library Preparation guide (Illumina Technical Note 15044223, no date). We obtained the taxonomic composition of the bacterial communities by analyzing the V3 E V4 region of the 16S rRNA gene using the Illumina^®^ MiSeq platform.

The constructions of the DNA sequencing libraries were performed according to the manufacturer’s instructions (Illumina, San Diego, CA, USA) and followed the same flow described by Caporaso et al. (2012) and Yatsunenko et al. (2012) (63, 64). Using 300 bp paired readings, and MiSeq v3 reagents, the ends of each reading are overlaid to generate complete high-quality readings from the V3 and V4 regions. More than 100,000 readings are generated per sample, commonly recognized as sufficient for metagenomic research. The fastq sequences were analyzed using the Illumina 16S Metagenomics software, which performs the taxonomic classification of the v3/v4 region of the 16S rRNA gene using the DADA2 database. All Metagenomics analysis included in the manuscript is available under request.

### 2.7 Statistical analysis

We described all statistical analyses and the number of variables in Figure Legends. For metabolic studies, we expressed data as mean ± standard error of the mean (SEM). We applied the Unpaired t-test two-tailed to compare distinct variables. One-way analysis of variance (One-way-ANOVA) was used to compare more than two groups of mice. Two-way ANOVA was applied to compare more than two mice groups in conjunction with the time effect. For One-way-ANOVA and Two-way ANOVA, we used the Bonferroni *post hoc* test. We used the statistical software GraphPad Prism 7.0. Software (San Diego, CA, USA) to analyze all data. P<0.05 were considered significant and described in the Results section and the Figure Legends. We performed data paired abundance analysis for the Gut Microbiota analysis using the IBM SPSS^®^ 20.0 software (Wilcoxon Signed Ranks Test). The alpha and beta diversity statistical analysis was performed using the EZbioCloud software. The graphs were generated by GraphPad Prism 7.0. Software (San Diego, CA, USA). P<0.05 was considered statistically significant.

## 3 Results

### 3.1 Maternal exposure to PM_2.5_ reprograms the energy balance of adult male and female offspring

We recorded the birth weight (g) of males and females together from the offspring. We did not observe statistical differences in the birth weight among the three groups (mean ± SEM: FA/FA: 1.24 g ± 0.03, n=6; PM_2.5_/FA: 1.40 g ± 0.04, n=17; FA/PM_2.5_: 1.41 g ± 0.04, n=8. One Way ANOVA: PM_2.5_/FA vs. FA/FA p=0.08) (**Supplemental Figure 6A**). We separated males and females upon weaning.

**Figure 1:**
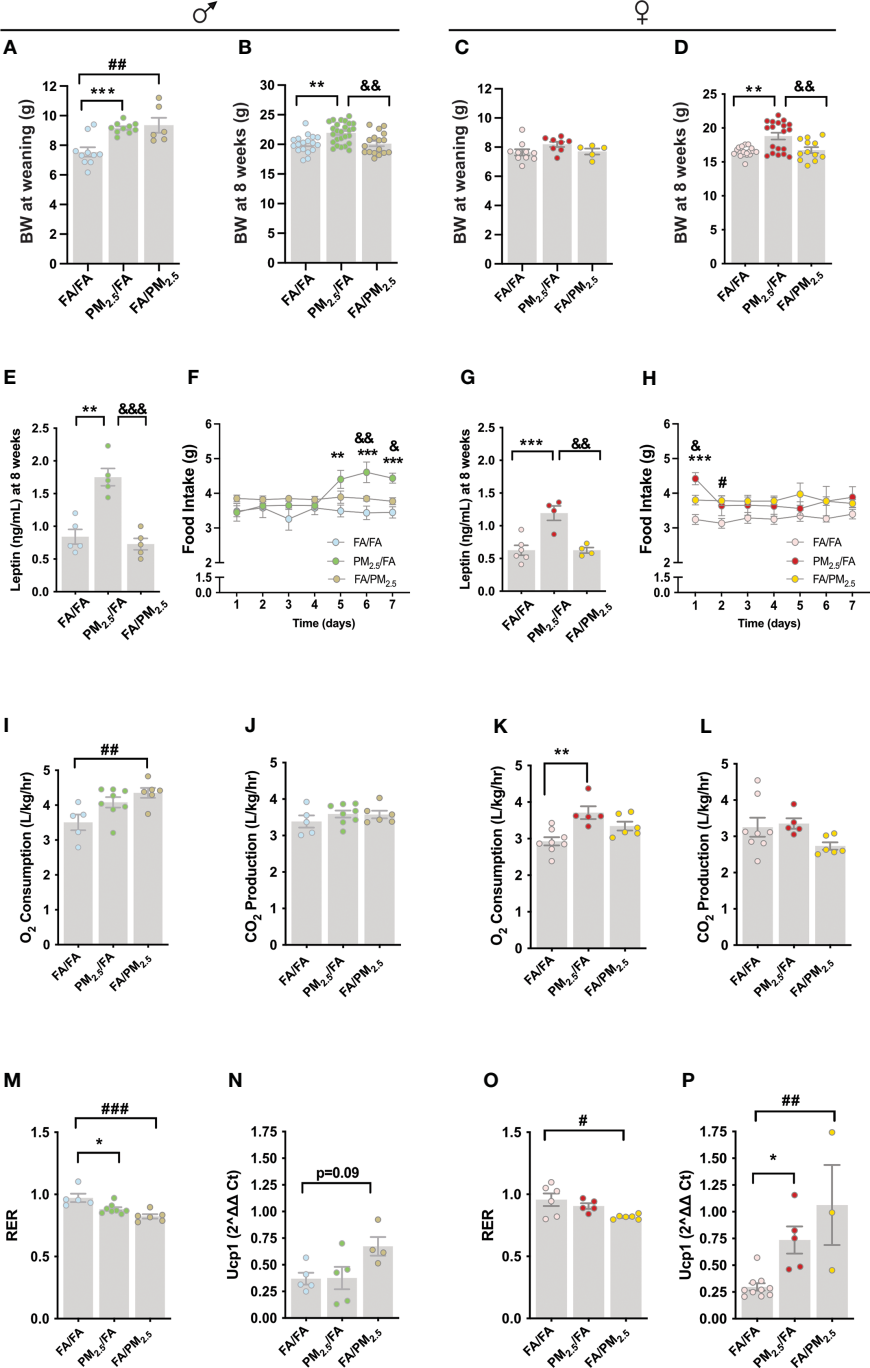
Maternal exposure to PM_2.5_ alters the energy metabolism of the offspring. **(A)** Male offspring body weight (g) at weaning, n=6-10. **(B)** Male offspring body weight (g), n=17-26. **(C)** Female offspring body weight (g) at weaning, n=5-10. **(D)** Female offspring body weight (g), n=12-19. **(E)** Male offspring fasting serum leptin level (ηg/mL), n=5 for each group. **(F)** Food intake (g), n=5 for each group. (g) Female offspring fasting serum leptin level (ηg/mL), n=3-6 for each group. **(H)** Female offspring food intake (g), n=5 for each group. **(I)** Male offspring oxygen (O2) consumption (L/kg/hr), n=5-8. **(J)** Carbon dioxide (CO2) production (L/kg/hr), n=5-8. **(K)** Female offspring O2 consumption (L/kg/hr), n=5-8. L. CO2 production (L/kg/hr), n=5-8. M. Male offspring respiratory exchange ratio (RER), n=5-8. N. Uncoupling Protein 1 (Ucp1) gene expression in the brown adipose tissue (BAT), n=8-13. O. Female offspring RER, n=5-8. P. Ucp1 gene expression in the BAT, n=3-10 from female offspring. Offspring mothers were exposed to PM_2.5_ during pregnancy (PM_2.5_/FA) or lactation (FA/PM_2.5_) and filtered air during pregnancy and lactation (FA/FA) as a control group. Panels **(B, D, E**, **G**) mice were 8 weeks. Panels **(F)** and **(H)**, mice were 13-14 weeks. Panels **(I-P)**, mice were 18-19 weeks. Data were expressed as the mean ± SEM. One-way ANOVA was used to analyze Panels **(A–E, G)** and **(I-P)**. Two-way ANOVA was used for Panels **(F)** and **(H)** We used Bonferroni as a *post hoc* test and a significance of P<0.05. p values: ***P<0.001; **P<0.01; *P<0.05 (PM_2.5_/FA vs. FA/FA); &&&P<0.001; &&P<0.01; &P<0.05 (PM_2.5_/FA vs. FA/PM_2.5_); ###P<0.001; ##P<0.01; #P<0.05 (FA/PM_2.5_ vs. FA/FA).

At weaning, we observed an increased body weight in male offspring from mothers exposed to PM_2.5_ during gestation (PM_2.5_/FA) (P<0.001) or lactation (FA/PM_2.5_) (P<0.01) compared to the control group (FA/FA) (**Figure 1A**). Male offspring from mothers exposed to PM_2.5_/FA at eight weeks old displayed a significant increase in body weight compared to FA/FA (P<0.01) and FA/PM_2.5_ (P<0.01) groups (**Figure 1B**). Unlike males, the females did not display differences in body weight at weaning among the groups (**Figure 1C**). In contrast, female PM_2.5_/FA at eight weeks old displayed a significant increase in body weight compared to FA/FA (P<0.001) and FA/PM_2.5_ (P<0.01) groups (**Figure 1D**). Leptin levels were also increased in the PM_2.5_/FA group compared to FA/FA (P<0.01) and FA/PM_2.5_ (P<0.001) groups in both genders at 8 weeks (**Figures 1E, G**).

To get insight into the mechanism of increased body weight, we measured seven-day food intake in young mice. At this time point, male and female offspring kept similar body weights as we observed at 8 weeks (**Supplemental Figures 6D, E**). We observed an increased food intake from the fifth through seventh days in the male PM_2.5_/FA compared to the FA/FA (5th day P<0.01; 6th day P<0.001; 7th day P<0.001) and FA/PM_2.5_ (6th day P<0.01; 7th day P<0.05) (**Figure 1F**). In females, food intake was only mildly increased at the beginning of measurement in the PM_2.5_/FA compared to the FA/FA (P<0.001) and FA/PM_2.5_ (P<0.05). On the second day of measurement, we observed a significant increase in food intake in the FA/PM_2.5_ compared to FA/FA group (P<0.05) (**Figure 1H**).

Curiously, O_2_ consumption increased only in the male FA/PM_2.5_ group compared to FA/FA (P<0.01) (**Figure 1I**). CO_2_ production was similar among groups (**Figure 1J**). PM_2.5_/FA (P<0.05) and FA/PM_2.5_ (P<0.01) displayed decreased RER compared to the FA/FA group (**Figure 1M**). Despite increased O_2_ consumption in the male FA/PM_2.5_ group, Ucp1 expression in the brown adipose tissue (BAT) was slightly high (P=0.09) in the FA/PM_2.5_ group than in FA/FA (**Figure 1N**). In the female offspring, the PM_2.5_/FA group presented higher (P<0.01) levels of O_2_ consumption (**Figure 1K**) consistent with higher (P<0.05) Ucp1 expression (**Figure 1P**) in the BAT compared to FA/FA. CO_2_ production was similar among female groups (**Figure 1L**). FA/PM_2.5_ displayed decreased RER compared to the FA/FA group (P<0.05) (**Figure 1O**). We observed higher Ucp1 expression in the BAT of the female FA/PM_2.5_ (P<0.01) group than in FA/FA (**Figure 1P**).

### 3.2 Maternal exposure to PM_2.5_ alters offspring’s hypothalamic neuropeptides and inflammatory markers

**Figure 2:**
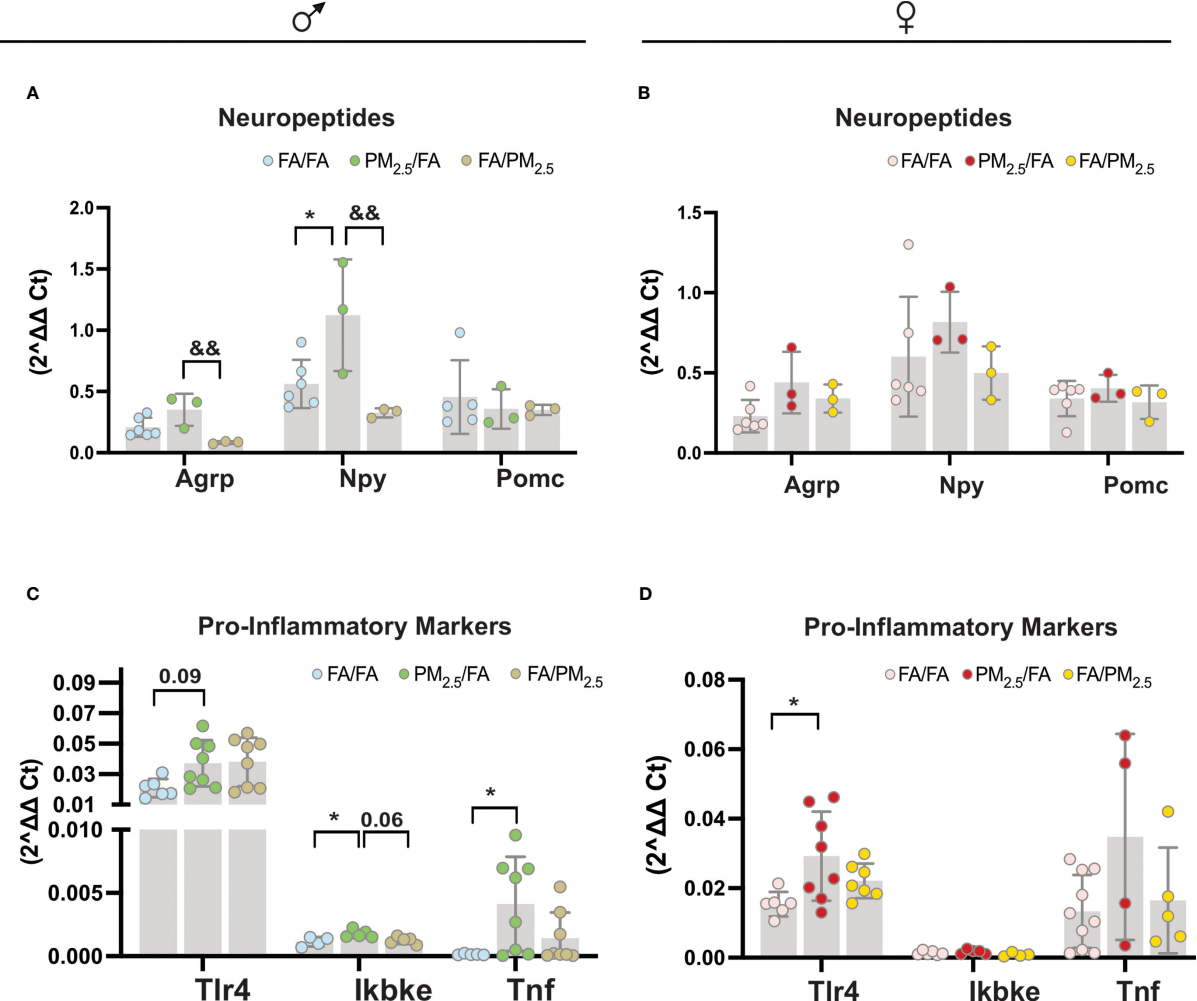
Maternal exposure to PM_2.5_ alters hypothalamic neuropeptide expression and pro-inflammatory signals significantly in the offspring. **(A)** (males): Agrp (Agouti-related protein); Npy (neuropeptide Y); and Pomc (Pro-opiomelanocortin) expression in the hypothalamus n=3-6. **(B)** (females): Agrp (Agouti-related protein); Npy (neuropeptide Y). And Pomc (Pro-opiomelanocortin) expression in the hypothalamus n=3-6. **(C)** (males): Tlr4 (Toll-like receptor 4); Ikbke and Tnf alpha (Tumor necrosis factor-alpha) expression in the hypothalamus n=4-8. **(D)** (females): Tlr4 (Toll-like receptor 4); ikke and Tnf alpha (Tumor necrosis factor-alpha) expression in the hypothalamus n=4-11 from offspring whose mothers were exposed to PM_2.5_ during pregnancy (PM_2.5_/FA) or lactation (FA/PM_2.5_) and filtered air during pregnancy and lactation (FA/FA) as a control group. All mice were 20 weeks of age. 2^delta Ct was used to determine each gene expression. Data were expressed as the mean ± SEM. One-way ANOVA was used for the statistical analysis. We used Bonferroni as a *post hoc* test and a significance of P<0.05. p values: *P<0.05 (PM_2.5_/FA vs. FA/FA); &&P<0.01 (PM_2.5_/FA vs. FA/PM_2.5_).

We observed an increase in the Npy gene expression in the hypothalamus of male offspring from the PM_2.5_/FA group compared to the FA/FA (P<0.05) and FA/PM_2.5_ (P<0.01) groups. Agrp gene expression was increased in the hypothalamus of male offspring from the PM_2.5_/FA group compared to the FA/PM_2.5_ (P<0.01) mice. We did not find differences in the Pomc gene expression in the hypothalamus among the three groups of male offspring (**Figure 2A**). For the females, we did not observe differences in the Agrp, Npy, and Pomc gene expression in the hypothalamus among the three groups (**Figure 2B**). Regarding the inflammatory markers, we detected a mild increase (P=0.09) in the Tlr4 gene expression in the hypothalamus of the male PM_2.5_/FA group compared to FA/FA. PM_2.5_/FA also displayed higher (P<0.05) levels of Ikkϵ compared to FA/FA and FA/PM_2.5_ (P=0.06). Tnf alpha gene expression was higher (P<0.01) in the hypothalamus of the PM_2.5_/FA group than in FA/FA (**Figure 2C**). Tlr4 gene expression was elevated (P<0.05) in the hypothalamus of the female PM_2.5_/FA group compared to FA/FA. We did not observe differences in the Ikbke and Tnf alpha gene expression in the hypothalamus among the three groups of females (**Figure 2D**).

### 3.3 Effect of maternal exposure to PM_2.5_ on gut microbiota

**Figure 3:**
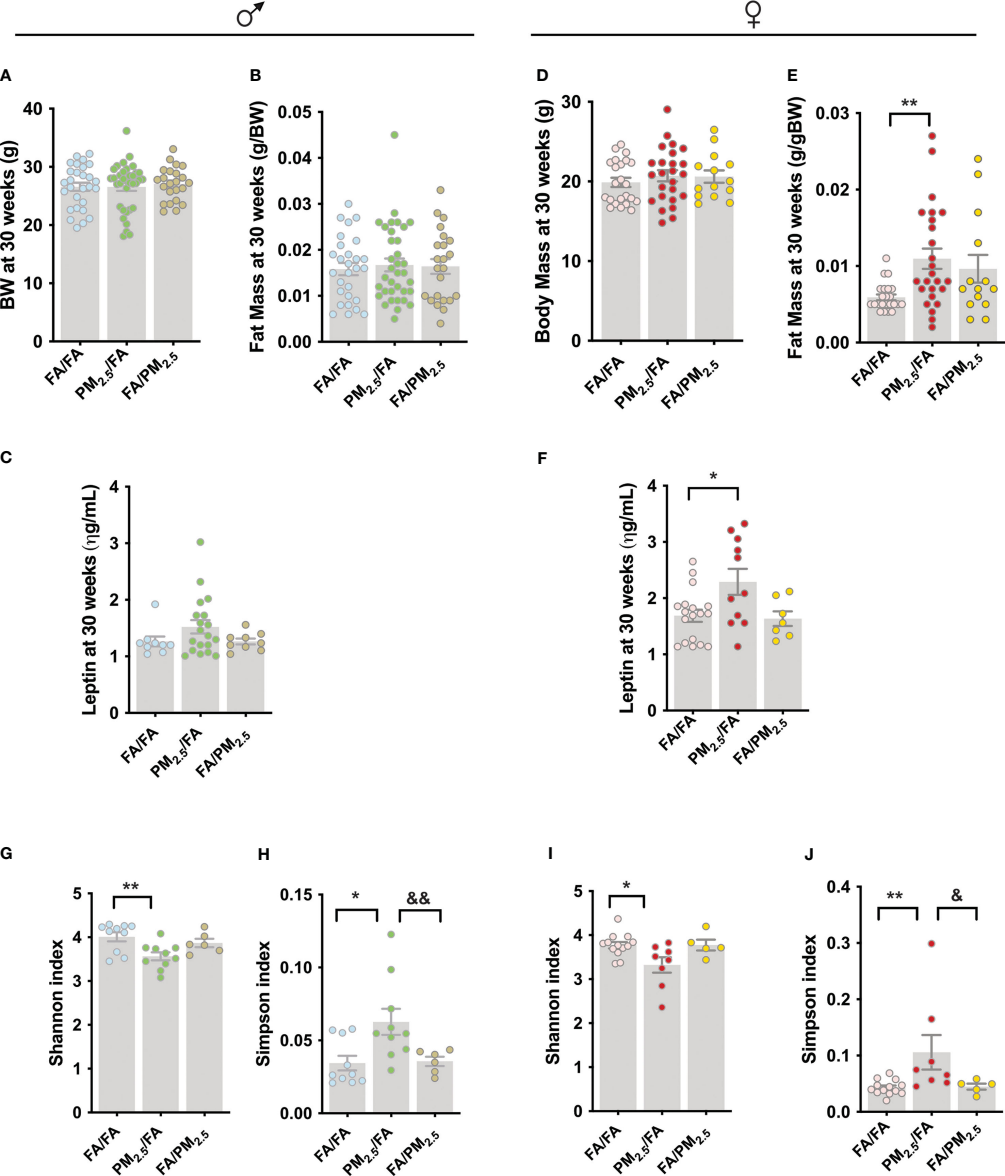
Maternal PM_2.5_ exposure alters alpha diversity and gut microbiota composition independent of gender. **(A)** Male body weight (g); **(B)** Male fat mass (g/gBW), n=23-35 and **(C)** Male fasting serum leptin level (ηg/mL), n=9-19. **(D)** Female body weight (g); **(E)** Female fat mass (g/gBW), n=14-26 and **(F)** Female fasting serum leptin level (ηg/mL), n=14-25. Alpha diversity was estimated by the **(G)** (male): Shannon index and **(H)** (male): Simpson index (n=6-10). Alpha diversity was estimated by the **(I)** (female): Shannon index and **(J)** (female): Simpson index i (n=5-13). **(A-C)** males and **(D-F)** females from offspring whose mothers were exposed to PM_2.5_ during pregnancy (PM_2.5_/FA) or lactation (FA/PM_2.5_) and filtered air during pregnancy and lactation (FA/FA) as a control group. All mice were 30 weeks of age. Data were expressed as the mean ± SEM. One-way ANOVA was used for the statistical analysis. We used Bonferroni as a *post hoc* test and a significance of P<0.05. p values: **P<0.01; *P<0.05 (PM2.5/FA vs. FA/FA); ^&&^P<0.01; ^&^P<0.05 (PM_2.5_/FA vs. FA/PM_2.5_).

In addition, to evaluate metabolic parameters in young offspring, we re-evaluated body weight, fat mass, leptin levels, and the gut microbiota in mice at 30 weeks. To our surprise, male offspring lost the difference in their body weight (**Figure 3A**). We found no differences in fat mass among the three groups (FA/FA, PM_2.5_/FA, and FA/PM_2.5_) (**Figure 3B**). These results were accompanied by no differences in serum leptin levels (**Figure 3C**). For female offspring, we did not observe differences in body weight (**Figure 3D**). However, fat mass (P<0.01) and leptin levels (P<0.05) were higher in the PM_2.5_/FA than in FA/FA group (**Figures 3E, F**).

We collected fecal samples from these mice and evaluated the gut microbiota diversity. More specifically, we investigate whether maternal exposure to PM_2.5_ alters the alpha and beta gut microbiota diversity. The analysis below showed the Shannon index, where a reduction indicates a decrease in alpha diversity, and the Simpsons index, where an increase implies a decrease in alpha diversity. Regarding the gut microbiota, the results demonstrated that male offspring from PM_2.5_/FA had a lower alpha diversity than FA/FA, indicated by the Shannon index (P<0.01) and by the Simpsons index (P<0.05). However, the lactation group (FA/PM_2.5_) had protection from this phenotype because the Simpsons index was lower (P<0.01) in this group compared to PM_2.5_/FA (**Figure 3G, H**).

Concerning females, the results are identical, with a reduction in alpha diversity in animals from the PM_2.5_/FA group than FA/FA using the Shannon index (P<0.05) and by Simpsons index (P<0.01). The FA/PM_2.5_ group had a lower Simpsons index (P<0.05) than PM_2.5_/FA, suggesting possible lactation protection for the lower alpha diversity induced by maternal pollution exposure (**Figures 3I, J**).

**Figure 4:**
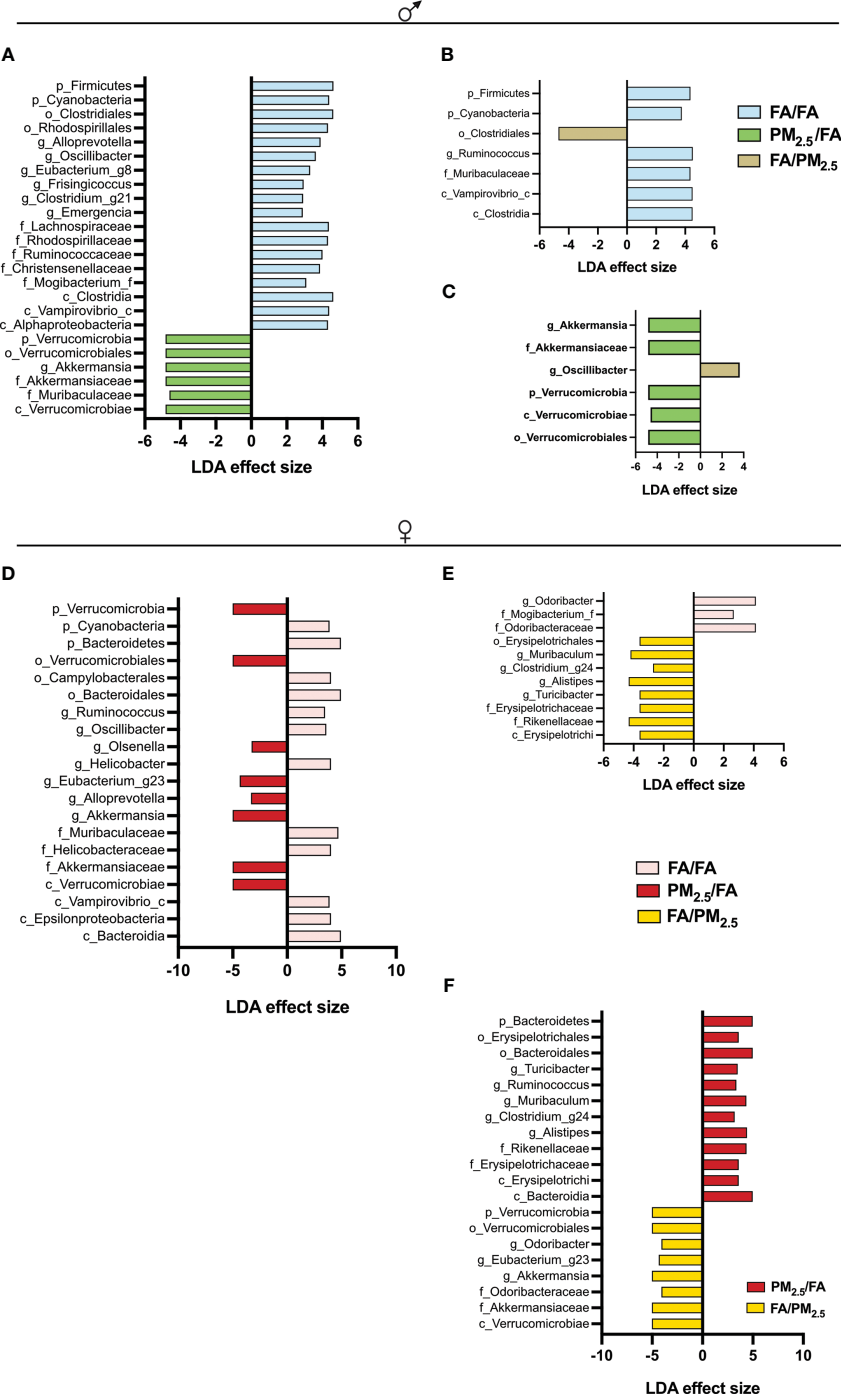
Maternal PM_2.5_ exposure alters the Linear discriminant analysis (LDA) effect size indicating differences in phyla and genera between **(A)** FA/FA and PM_2.5_/FA; **(B)** FA/FA and FA/PM_2.5_; **(C)** PM_2.5_/FA and FA/PM_2.5_ groups of males. LDA between **(D)** FA/FA and PM_2.5_/FA; **(E)** FA/FA and FA/PM_2.5_; **(F)** PM_2.5_/FA and FA/PM_2.5_ groups of females. Offspring mothers were exposed to PM_2.5_ during pregnancy (PM_2.5_/FA) or lactation (FA/PM_2.5_) and filtered air during pregnancy and lactation (FA/FA) as a control group. All mice were 30 weeks of age. The taxa with LDA score > 2 and significance of <0.05 were determined by the Wilcoxon signed-rank test.

Regarding beta diversity, in males, significant differences were between FA/FA and PM_2.5_/FA and PM_2.5_/FA versus FA/PM_2.5_, but not between FA/FA and FA/PM_2.5_ (**Figures 4A-C**). We observed a different beta diversity for females: control (FA/FA) against each of them and between the mothers exposed during gestation or lactation (**Figures 4D-F**).

**Figure 5:**
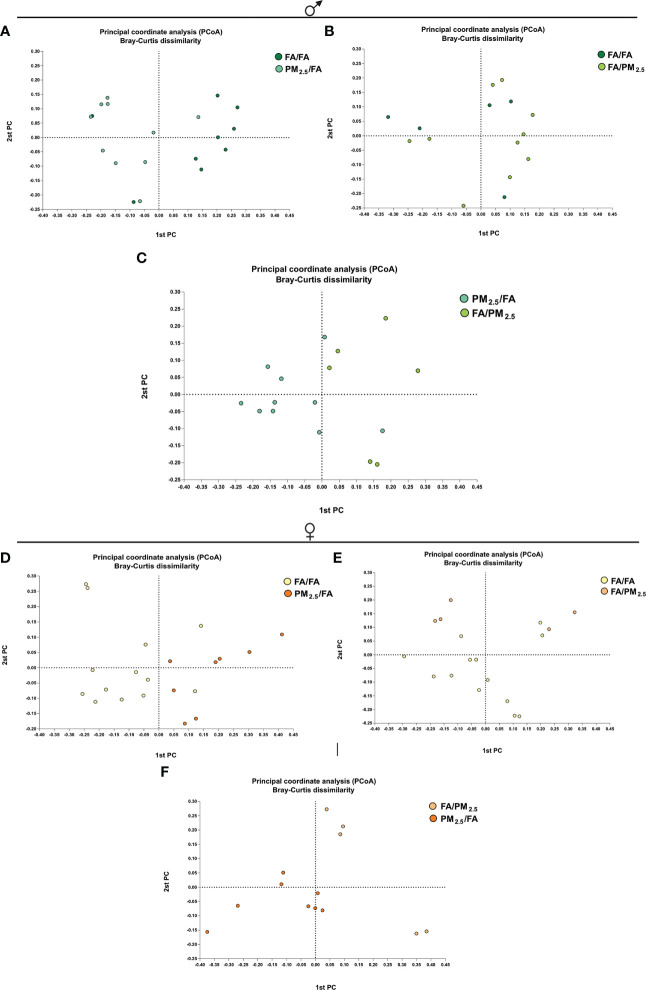
Principal coordinate analysis (PCoA) plot with Bray-Curtis dissimilarity. Ordination (PCoA) generated by using the Bray–Curtis dissimilarity metric sampled. Samples are colored according to the group. Male offspring **(A, B)** Bray-Curtis PCoA ordination. Results revealed that the FA/FA group displayed a spatial separation from PM_2.5_/FA but not from FA/PM_2.5_ (PERMANOVA: p = 0.007 and p = 0.46, respectively) **(C)** PM_2.5_/FA group showed a spatial separation from FA/PM_2.5_ (PERMANOVA: p = 0.004). Female offspring **(D, E)** Bray-Curtis PCoA ordination. Results revealed that the FA/FA group displayed a spatial separation from PM_2.5_/FA and FA/PM_2.5_ (PERMANOVA: p = 0.001 and p = 0.030, respectively). **(F)** PM_2.5_/FA group showed a spatial separation from FA/PM_2.5_ (PERMANOVA: p = 0.002).

The Bray-Curtis dissimilarity was used to compare the bacterial composition of microbiota among groups of male offspring. The FA/FA group displayed a spatial separation from PM_2.5_/FA but not from FA/PM_2.5_ (PERMANOVA: p = 0.007 and p = 0.46, respectively) (**Figures 5A, B**). PM_2.5_/FA group showed a spatial separation from FA/PM_2.5_ (PERMANOVA: p = 0.004) (**Figure 5C**).

The Bray-Curtis dissimilarity was used to compare the bacterial composition of microbiota among groups of female offspring. FA/FA group displayed a spatial separation from PM_2.5_/FA and FA/PM_2.5_ (PERMANOVA: p = 0.001 and p = 0.030, respectively) (**Figures 5D, E**). PM_2.5_/FA group showed a spatial separation from FA/PM_2.5_ (PERMANOVA: p = 0.002) (**Figure 5F**).

## 4 Discussion

We demonstrated that maternal exposure to air pollution affects the offspring’s energy metabolism. To our surprise, these findings were time-dependent throughout the offspring’s life and gender-specific.

Birth weight is a parameter for estimating intrauterine growth (65, 66). Our study found that PM_2.5_ exposure during gestation was associated with a tendency to increase the offspring’s birth weight independently of the gender, which is compatible with increased fetus growth. Males in the PM_2.5_/FA group displayed increased body weight from weaning until young adulthood. However, this difference diminished at 30 weeks of age. Nonetheless, the females in the PM_2.5_/FA group did not display higher body weight at weaning. However, the females were heavy at a young age (8 weeks) and kept higher adiposity until later in life (30 weeks). This finding suggests that the effect of PM_2.5_ during gestation was long-lasting only in females. Several studies demonstrated that PM_2.5_ exposure during gestation affects body weight. However, some studies showed the opposite of us, a reduction in birth body weight (49, 50). Even though these mice evolved with increased body weight in adulthood (49, 50), another study showed an unchanged offspring birth weight and a decrease in adulthood (48).

The difference between our results might be due to different time points of maternal exposure to PM_2.5_. Differently from us, other studies (48–50) exposed the females during gestation and seven weeks before. The three studies mentioned above suggested maternal preconception exposure might be more harmful than during gestation (48–50). Thus, the mild phenotype we observed in our study might be partially due to limited time points of exposure. The inconsistency among studies may also rely on the route of administration of PM_2.5_ and the source of PM_2.5_. Some studies employed intratracheal liquid instillation (48). Other studies (49, 50) and ours used concentrated PM_2.5_ in specialized chambers. The sources of pollutants might also contribute to different results since the composition of PM_2.5_ depends on the emission sources.

Even though other studies found lower birth weight after maternal PM_2.5_ exposure, the outcome was increased body weight at a younger age, at least for male offspring. This result agrees with our data that showed higher body weight in male offspring whose mothers were exposed to PM_2.5_ either during gestation or lactation. Together these data suggest that changes in birth weight, whether low or high, induce weight gain in young male adulthood. In female offspring, body weight at weaning was similar among the three groups. However, they displayed an increase in body weight at a younger age. Adipose tissue delivers leptin balancing with triglyceride stores (67, 68). Thus, circulating leptin is known to define body energy stores (67–69). We found increased leptin levels in the same groups with elevated body weight in younger offspring. Later in life, only females displayed high leptin levels and gonadal fat mass. Concurrently the increased leptin levels suggested increased fat mass.

Our data showed an elevation of food intake followed by increased levels of the orexigenic Agrp and Npy in males from the PM_2.5_/FA group compared to FA/FA and FA/PM_2.5_. The elevated Agrp and Npy expression in the hypothalamus might count for the increased food intake we observed in this group. In the arcuate nucleus, the central melanocortin system comprises Pomc, Agrp, and melanocortin receptors (MC4Rs). Human mutations in the MC4R trigger obesity (70), and AgRP neurons are the natural antagonist of these receptors favoring increased food intake (8). Changes in maternal nutrition alter the innervation of Pomc and Agrp in arcuate to PVN. The critical window to compromise these projections is during lactation, not gestation. This occurred since rodent projections are developed three weeks after the offspring is born. In humans, the development of those projections occurs *in utero* (8, 15–18, 71). Lippert et al. (2020) demonstrated that maternal exposure to HFD during lactation revealed sexually dimorphic expression of dopamine phenotypes, males showing hyperlocomotion, and females displaying a high preference for sucrose (15). Another study exhibited that maternal HFD exposure seven days before and during gestation and lactation predisposes the offspring to develop insulin resistance and obesity by activating white adipose tissue inflammatory pathways (72).

In contrast, maternal undernutrition also increases the risk of the offspring developing metabolic diseases such as obesity (73). In male offspring from the FA/PM_2.5_ group, despite displaying elevated body weight at weaning, the body weight was similar to the FA/FA group at eight weeks of age. The leptin levels followed decreased body weight, suggesting a decreased fat mass (67, 68) in this group.

Air pollution exposure, as well as changes in nutritional diet, have an impact on energy balance. In our study, female and male offspring from FA/PM_2.5_ group displayed higher RER. This result indicates changes in substrate utilization, burning more fat than carbohydrates. The level of O_2_ consumption and Ucp1 expression in BAT was not uniform in our data. Another study that exposed the mothers to PM_2.5_ observed decreased Ucp1 expression in BAT in the group PM_2.5_-exposed during lactation (48). This result was accompanied by increased adiposity (48). In males, O_2_ consumption was higher in offspring from FA/PM_2.5_ group than FA/FA, but Ucp1 expression showed a tendency to increase but was not significant. Interestingly, this offspring (from the FA/PM_2.5_ group) displayed lower body weight and leptin levels than PM_2.5_/FA group, suggesting that higher O_2_ consumption might contribute to decreasing body mass in this group. Together with a mild increase in food intake looks like maternal exposure only during lactation had a lower effect on the EE of male offspring. Females from the PM_2.5_/FA group also displayed higher O_2_ consumption and Ucp1 in the BAT than FA/FA. However, despite higher EE, PM_2.5_/FA females presented higher body weight and leptin levels. Thus, in this group, the increased food intake and the RER might contribute to keeping the higher body weight than the EE.

We also observed that maternal exposure to PM_2.5_ during pregnancy reduces the gut microbiota diversity and changes the composition independently of the offspring’s gender. However, to our surprise, the body weight, fat mass, and leptin levels in males lost the difference among the three groups. On the contrary, we observed an increased fat mass and leptin levels without changes in body weight in the female PM_2.5_/FA group than in FA/FA.

Evidence demonstrated that exposure to air pollution affects gastrointestinal health (74, 75); however, the results are conflicting. Kim et al. (2014) showed that exposure to air pollution reduces gut microbiota biodiversity (74). In contrast, Mutlu et al. (2019) suggested that PM_2.5_ exposure increases gut microbiota alpha diversity (39, 75). Also, several studies (76–78) highlighted the influence of air pollution on obesity and T2DM through gut microbiota. Our study observed that maternal PM_2.5_ exposure displayed altered offspring gut microbiota composition, characterized by changes in bacterial diversity and different amounts of some taxon. Similar to other studies (78–80), our results showed that the male and female offspring PM_2.5_/FA group displayed decreased alpha diversity (Shannon or Simpson index) compared to the FA/FA and the FA/PM_2.5_ groups.

In contrast, Liu et al. (2020) described an increase in alpha diversity associated with maternal PM_2.5_ exposure during the gestational period (77). In the same study, despite an increase in alpha diversity, beta diversity analysis revealed an apparent dissociation between the control and PM_2.5_ exposed group (77). Our study also observed an evident dissociation among the three groups. We observed differences in the abundance of some taxon among the groups. Female PM_2.5_/FA offspring showed lower proportions of Bacteroidetes phylum and higher proportions of Verrucomicrobia compared to female FA/FA or FA/PM_2.5_. Like females, male PM_2.5_/FA offspring showed higher ratios of Verrucomicrobia phylum but lower proportions of Firmicutes phylum compared to male FA/FA or FA/PM_2.5_. In our study, more significant amounts of Akkermansia (Verrucomicrobia phylum) were observed in the male and female offspring from the PM_2.5_/FA group compared to FA/FA group. Akkermansia has been associated with improved glucose homeostasis and weight loss (41, 77, 81, 82). It is known that genetic and environmental factors can change microbiota composition, and if the individual is obese, the changes could be more dramatic. It is also essential to mention that the gut microbiota composition can variate within an individual (41, 83).

Serino et al. (2012) described that an increased Allistipes genus could be a protective factor to type 2 diabetes in an HFD scenario (84). A study by our group (83) demonstrated that probiotic administration in DIO animals could increase these genera. Unexpectedly, our study observed increased amounts of Allistipes in females FA/PM_2.5_ compared to FA/FA and PM_2.5_/FA compared to FA/PM_2.5_. This result could also explain the less harmful effect of maternal exposure to PM_2.5_.

Thingholm et al. (2019) observed a significant association between some individual microbial genera with obesity, pointing to the decrease in Akkermansia, Oscillibacter, and Allistipes (85). There were similarities in our results with this mentioned study. We also observed increased amounts of Oscillibacter in males FA/FA and FA/PM_2.5_ compared to PM_2.5_/FA. Still, our data differed slightly from Thingholm et al. (2019) when analyzing Akkermansia and Allistipes, as already discussed above (85).

However, some studies demonstrate that excessive mucin degradation can favor the access of pathogens to the mucosa (86, 87). Polluting particles can affect the protective characteristics of the mucus in the intestinal tract, changing the permeability and composition of the microbiota and impairing health (88). Although the offspring have not been subject to pollution, maternal exposure during gestation can result in placental epigenetic modification and fetus reprogramming (89).

The current study has some limitations regarding data collection time points. We measured food intake at 13-14 weeks and energy expenditure at 18-19 weeks. Nevertheless, the body weight at 13 weeks kept a similar pattern as 8 weeks in both genders, not representing a critical interpretation problem. In the current study, we evaluated gut microbiota composition only from mice at 30 weeks. Surprisingly, male but not female offspring lost the difference in their body weight and serum leptin levels. On the contrary, in another study (48), only male offspring but not female offspring from mothers exposed to CAP displayed increased adiposity in adulthood. However, in this other study, all mothers were exposed during pre-conception (48).

In summary, our data support the hypothesis that exposure to air pollution during gestation is more harmful to energy balance than exposure during lactation, independent of gender. Male offspring had an unfavorable metabolic phenotype at a young age. However, at an older age, only females kept more adiposity. Ultimately, our data highlights the importance of controlling air pollution, especially during gestation.

## Data availability statement

The original contributions presented in the study are publicly available. This data can be found here: https://www.ncbi.nlm.nih.gov/bioproject/PRJNA894691. In addition, we included some raw data in the **Supplementary Material**. Further inquiries can be directed to the corresponding author.

## Author contributions

PP, MV, AS conceived the study, designed experiments, and interpreted the results. OZ, CC, VY, AS, GC, CF, SN, and PP performed all experiments. PP wrote the manuscript. OZ, CC, Y-BK, AS, MV, PS, and MS helped with discussions and edited the manuscript. All authors contributed to the article and approved the submitted version.
