# Supplementary figures and images for: Maternal exposure to air pollution alters energy balance transiently according to gender and changes gut microbiota
Source: Front Endocrinol (Lausanne). 2023 Apr 4;14:1069243. doi: 10.3389/fendo.2023.1069243 (PMC10112381; doi:10.3389/fendo.2023.1069243)

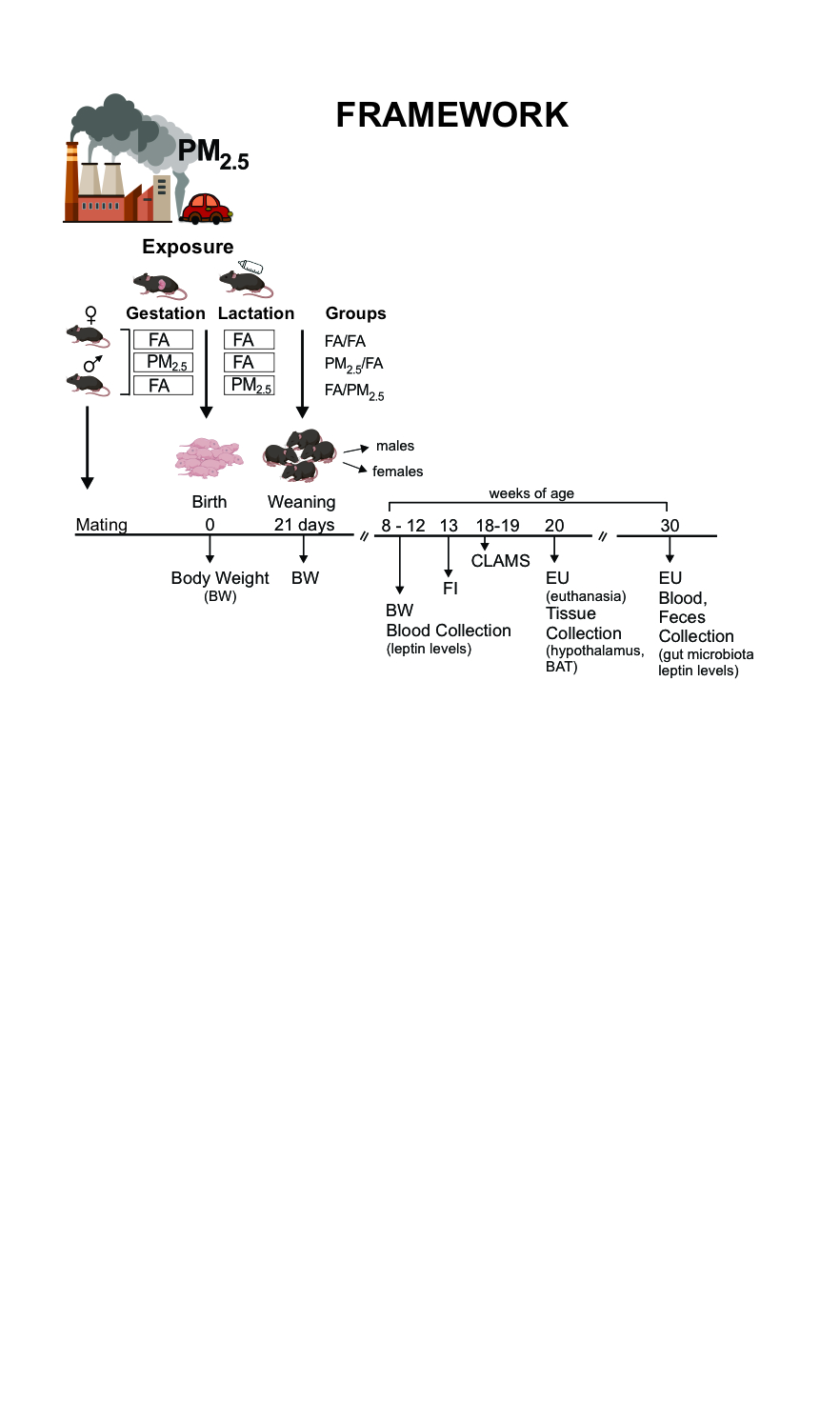

Supplement: Supplementary file 1 [file Image_1.jpg]
