# Supplementary material for: Maternal exposure to air pollution alters energy balance transiently according to gender and changes gut microbiota
Source: Front Endocrinol (Lausanne). 2023 Apr 4;14:1069243. doi: 10.3389/fendo.2023.1069243 (PMC10112381; doi:10.3389/fendo.2023.1069243)
Supplement: Supplementary file 2 [file DataSheet_1.pdf]

Supplemental Figure

Birth Weight		
FA/FA	$PM_{2.5}$/FA	FA/$PM_{2.5}$
1.33	1.32	1.52
1.27	1.37	1.29
1.32	1.62	1.51
1.18	1.68	1.45
1.20	1.80	1.25
1.15	1.41	1.42
	1.39	1.51
	1.43	1.37
	1.26	
	1.55	
	1.38	
	1.16	
	1.24	
	1.33	
	1.41	
	1.19	
	1.25	

Males Data

Fig 1A		
FA/FA	$PM_{2.5}$/FA	FA/$PM_{2.5}$
6.87	9.44	8.70
6.16	9.13	8.40
7.38	9.85	8.90
7.33	9.43	10.60
6.85	8.53	11.20
7.60	9.04	8.31
7.30	9.17	
9.10	8.83	
7.50	9.28	
9.40	8.90	

Fig 1E		
FA/FA	$PM_{2.5}$/FA	FA/$PM_{2.5}$
1.20	2.23	0.60
1.00	1.61	0.72
0.70	1.78	1.00
0.70	1.70	0.50
0.60	1.44	0.82

FIG 1 I		
FA/FA	$PM_{2.5}$/FA	FA/$PM_{2.5}$
3.225769231	4.037857143	4.827619048
3.726250000	4.452040816	4.381785714
4.091250000	3.956632653	4.419642857
3.691666667	4.266683673	4.295595238
2.789583333	3.204693878	3.748452381
	4.452500000	4.442976190
	4.405459184	
	3.878418367	

FIG 1J		
FA/FA	$PM_{2.5}$/FA	FA/$PM_{2.5}$
3.011730769	3.671275510	4.029523810
3.556250000	3.876428571	3.577380952
3.923333333	3.364387755	3.434047619
3.367291667	3.593316327	3.383333333
3.076458333	3.108928571	3.363809524
	3.862551020	3.671785714
	3.808724490	
	3.430816327	

Fig 1B		
FA/FA	$PM_{2.5}$/FA	FA/$PM_{2.5}$
19.90	24.14	23.11
20.29	20.84	20.68
19.77	21.47	20.74
19.01	21.02	21.80
19.18	22.66	18.66
21.68	24.12	18.54
20.97	22.28	18.19
21.25	22.41	19.72
20.75	23.30	23.30
21.20	23.97	18.55
23.57	23.88	18.70
20.55	23.50	18.72
19.06	22.60	17.62
18.63	22.90	21.14
17.32	23.50	22.63
17.59	24.75	20.71
19.98	21.68	19.16
20.23	20.15	
	18.98	
	19.65	
	22.15	
	19.22	
	19.50	
	21.32	
	19.73	
	20.36	

FIG 1 M		
FA/FA	$PM_{2.5}$/FA	FA/$PM_{2.5}$
0.93365	0.90921	0.83468
0.95438	0.87071	0.81642
0.95896	0.85032	0.77700
0.91213	0.84218	0.78763
1.10284	0.97012	0.89739
	0.86750	0.82642
	0.86455	
	0.88459	

FIG 1N		
FA/FA	$PM_{2.5}$/FA	FA/$PM_{2.5}$
0.313709	0.456125	0.614933
0.567031	0.426909	0.514413
0.411083	0.130173	0.924343
0.307147	0.160985	0.639050
0.249740	0.700036	

FIG 3A		
FA/FA	$PM_{2.5}$/FA	FA/$PM_{2.5}$
30.73	28.93	26.60
31.25	27.02	23.46
28.55	30.12	33.06
30.49	26.32	29.44
27.29	28.02	27.45
26.48	27.15	26.02
24.83	28.04	23.62
25.78	27.84	22.30
26.29	27.60	24.21
26.58	27.76	23.54
26.28	20.17	22.46
27.72	22.60	25.74
22.90	21.11	29.26
22.62	18.07	27.26
21.10	18.84	26.32
22.50	18.36	28.09
20.87	28.45	27.13
20.32	28.55	27.77
19.51	22.90	28.15
23.90	27.90	28.85
29.00	28.45	30.17
29.95	23.15	30.42
29.22	22.20	31.27
27.48	22.56	
28.21	29.63	
31.83	29.15	
30.51	30.70	
32.25	27.25	
	31.75	
	36.17	
	30.15	
	29.62	
	27.19	
	27.12	
	28.97	

FIG 3B		
FA/FA	$PM_{2.5}$/FA	FA/$PM_{2.5}$
0.019	0.017	0.018
0.021	0.028	0.023
0.019	0.026	0.021
0.023	0.026	0.027
0.03	0.025	0.025
0.027	0.026	0.019
0.027	0.022	0.028
0.026	0.024	0.033
0.016	0.045	0.018
0.018	0.026	0.014
0.015	0.022	0.022
0.015	0.017	0.021
0.022	0.019	0.016
0.018	0.025	0.016
0.018	0.018	0.004
0.017	0.016	0.009
0.013	0.013	0.010
0.016	0.012	0.008
0.006	0.011	0.007
0.006	0.011	0.009
0.006	0.014	0.009
0.012	0.015	0.010
0.008	0.015	0.010
0.007	0.013	
0.008	0.008	
0.011	0.009	
0.009	0.005	
0.01	0.010	
	0.009	
	0.008	
	0.007	
	0.011	
	0.012	
	0.011	
	0.009	

FIG 3 C		
FA/FA	$PM_{2.5}$/FA	FA/$PM_{2.5}$
1.0400	1.0400	1.1047
1.0723	1.2018	1.0400
1.2342	1.0077	1.5598
1.2018	1.5598	1.1694
1.2342	1.9537	1.3317
1.9208	1.2018	1.3317
1.2018	1.0723	1.2018
1.1694	1.7235	1.2342
1.2992	3.0210	1.3967
	1.3642	
	1.2667	
	2.3178	
	2.0197	
	1.7235	
	1.0077	
	1.5925	
	1.4619	
	1.1047	
	1.2992	

FIG 3 G		
FA/FA	$PM_{2.5}$/FA	FA/$PM_{2.5}$
4.258926	3.395463	3.820396
4.292982	3.637339	3.698982
4.135840	3.761081	3.591419
4.232169	3.235548	4.241640
4.106536	3.453945	4.024140
3.452820	3.075785	3.833426
3.664874	4.080848	
4.194495	3.756763	
3.523704	3.513796	
4.253581	3.733355	

FIG 3H		
FA/FA	$PM_{2.5}$/FA	FA/$PM_{2.5}$
0.020855	0.071744	0.042314
0.021209	0.054671	0.034883
0.026793	0.040212	0.043602
0.022300	0.098591	0.024062
0.033081	0.058733	0.028569
0.057061	0.122717	0.040368
0.055167	0.029614	
0.026246	0.052150	
0.057874	0.054857	
0.023970	0.043916	

FIG 4 A	
c_Verrucomicrobiae	-4.83025
f_Muribaculaceae	-4.61178
f_Akkermansiaceae	-4.83025
g_Akkermansia	-4.83025
o_Verrucomicrobiales	-4.83025
p_Verrucomicrobia	-4.83025
c_Alphaproteobacteria	4.31356
c_Vampirovibrio_c	4.38895
c_Clostridia	4.61738
f_Mogibacterium_f	3.09461
f_Christensenellaceae	3.87055
f_Ruminococcaceae	4.00361
f_Rhodospirillaceae	4.31356
f_Lachnospiraceae	4.36480
g_Emergencia	2.89099
g_Clostridium_g21	2.91367
g_Frisingicoccus	2.94341
g_Eubacterium_g8	3.31533
g_Oscillibacter	3.63143
g_Alloprevotella	3.89637
o_Rhodospirillales	4.31356
o_Clostridiales	4.61738
p_Cyanobacteria	4.38895
p_Firmicutes	4.62021

FIG 4 B	
c_Clostridia	4.50939
c_Vampirovibrio_c	4.50939
f_Muribaculaceae	4.35865
g_Ruminococcus	4.51101
o_Clostridiales	-4.7023
p_Cyanobacteria	3.76148
p_Firmicutes	4.35865

FIG 4 C	
o_Verrucomicrobiales	-4.83025
c_Verrucomicrobiae	-4.61178
p_Verrucomicrobia	-4.83025
g_Oscillibacter	3.63143
f_Akkermansiaceae	-4.83025
g_Akkermansia	-4.83025

Fig 1F

FA/FA					PM2.5/FA				
3.98	2.99	3.54	3.53	3.37	4.38	2.94	3.51	3.37	3.06
3.90	2.80	4.49	3.40	3.34	4.04	3.29	3.67	3.59	3.62
4.00	3.20	2.10	3.56	3.43	4.05	3.30	3.68	3.60	3.64
3.98	2.99	4.04	3.53	3.36	4.04	3.28	3.73	3.59	3.59
4.02	2.97	3.50	3.60	3.37	5.39	3.83	4.33	4.26	4.21
3.98	2.76	3.49	3.55	3.41	5.33	3.67	4.89	4.21	4.93
4.02	3.01	3.35	3.50	3.37	4.52	4.01	4.84	4.25	4.57

FA/PM2.5				
3.56	4.01	3.89	3.71	4.09
3.50	3.98	3.87	3.68	4.07
3.57	3.99	3.89	3.70	4.09
3.55	3.99	3.87	3.65	4.03
3.30	4.19	3.85	3.81	4.31
3.70	3.79	3.91	3.75	4.08
3.56	3.95	3.88	3.50	3.97

Fig 2A

	FA/FA						PM2.5/FA		
AgRP	0.148137	0.159596	0.3259	0.145038	0.285191	0.181999	0.199921	0.413942	0.438303
NPY	0.562139	0.462813	0.901563	0.409518	0.663883	0.373842	0.645281	1.169993	1.553476
POMC	0.252525	0.380113	0.393518	0.268874	0.981119		0.247586	0.283123	0.542745

	FA/PM2.5		
AgRP	0.093266	0.072193	0.087141
NPY	0.339739	0.354904	0.282731
POMC	0.358986	0.303759	0.385419

Fig 2C								
	FA/FA							
TLR4	0.014195	0.030937	0.022305	0.017283	0.017116	0.023462		
ikke	0.001001	0.001489	0.000692	0.001405				
TNFα	0.000103	0.000237	0.000129	0.000144	0.0000957			
	$PM_{2.5}$/FA							
TLR4	0.020454	0.021226	0.026406	0.04042	0.06168	0.02838	0.05008	0.04831
ikke	0.001905	0.001545	0.001506	0.002253	0.001603			
TNFα	0.006956	0.006896	0.006202	0.000496	0.000153	0.009578	0.002697	0.0000571
	FA/$PM_{2.5}$							
TLR4	0.018186	0.020667	0.021367	0.04977	0.05243	0.03713	0.04773	0.05682
ikke	0.001081	0.000869	0.00137	0.001602	0.001315			
TNFα	0.003478	0.005467	0.001743	0.000224	0.000144	0.0000562	0.0000585	0.000135

Females _Data

Fig 1C		
FA/FA	$PM_{2.5}$/FA	FA/$PM_{2.5}$
7.59	7.91	7.00
7.28	7.25	7.97
7.63	7.81	7.49
7.26	8.40	7.95
7.67	8.32	8.10
6.70	8.48	
7.20	8.62	
7.70	8.75	
8.20		
9.20		

Fig 1G		
FA/FA	$PM_{2.5}$/FA	FA/$PM_{2.5}$
0.54	1.31	0.65
0.71	0.87	0.54
0.41	1.36	0.58
0.94	1.23	0.73
0.63		
0.52		

Fig 1K		
FA/FA	$PM_{2.5}$/FA	FA/$PM_{2.5}$
3.03694	3.33500	3.73677
3.42582	4.37488	3.68147
3.25496	3.61579	3.02568
2.89307	3.62404	3.29738
2.71288	3.59261	3.17772
2.84923		3.14386
2.38346		
2.86365		

Fig 1L		
FA/FA	$PM_{2.5}$/FA	FA/$PM_{2.5}$
3.11557	3.22988	3.08031
2.81115	3.89056	3.02272
3.07519	3.20625	2.56340
2.31615	3.05202	2.62965
2.85115	3.38534	2.60000
3.12631		2.50443
4.54517		
4.17710		

Fig 1D		
FA/FA	$PM_{2.5}$/FA	FA/$PM_{2.5}$
16.03	21.03	19.01
16.11	19.76	16.45
17.57	19.51	16.06
16.81	20.52	15.25
16.35	20.35	16.2
14.67	20.93	15.75
16.2	20.04	17.85
16.07	21.14	18.40
16.55	21.88	14.44
15.89	20.77	15.12
17.13	16.90	18.61
17.30	16.94	17.73
17.59	16.25	
16.46	15.72	
16.59	16.01	
16.25	15.87	
16.46	16.13	
17.02	17.26	
	20.15	

Fig 1O		
FA/FA	$PM_{2.5}$/FA	FA/$PM_{2.5}$
1.02589	0.96848	0.82432
0.82058	0.88930	0.82106
0.94477	0.88673	0.84721
0.80058	0.84216	0.79749
1.05097	0.94231	0.81819
1.09725		0.79660

Fig 1P		
FA/FA	$PM_{2.5}$/FA	FA/$PM_{2.5}$
0.338564	0.462011	1.742308
0.570777	1.155486	0.452817
0.354289	0.824162	0.992748
0.248273	0.751841	
0.270878	0.486496	
0.284838		
0.209964		
0.267056		
0.206715		
0.222938		

Females _Data

Fig 3D		
FA/FA	$PM_{2.5}$/FA	FA/$PM_{2.5}$
22.35	21.05	21.39
22.14	19.95	21.91
22.38	22.18	25.28
21.78	21.31	22.14
22.69	22.22	20.04
24.15	22.30	17.20
24.64	22.35	17.30
22.65	24.40	18.10
23.64	14.80	18.20
19.78	17.67	18.92
20.23	18.20	19.36
16.40	19.02	19.10
19.67	19.28	23.20
17.15	18.15	26.48
17.19	20.92	
18.02	15.38	
18.20	16.30	
16.71	29.03	
17.71	20.80	
17.50	16.67	
17.90	17.54	
17.80	24.14	
16.70	25.73	
	24.70	
	23.56	

Fig 3E		
FA/FA	$PM_{2.5}$/FA	FA/$PM_{2.5}$
0.007	0.017	0.022
0.004	0.017	0.017
0.007	0.025	0.024
0.004	0.015	0.007
0.005	0.027	0.008
0.011	0.016	0.013
0.009	0.019	0.008
0.009	0.017	0.007
0.005	0.010	0.007
0.007	0.008	0.006
0.005	0.011	0.005
0.005	0.006	0.005
0.004	0.008	0.003
0.006	0.009	0.003
0.005	0.013	
0.005	0.008	
0.005	0.003	
0.007	0.005	
0.006	0.004	
0.005	0.007	
0.006	0.008	
0.005	0.002	
0.004	0.005	
	0.007	
	0.007	

Fig 3J		
FA/FA	$PM_{2.5}$/FA	FA/$PM_{2.5}$
0.05967	0.065485	0.058374
0.037059	0.089117	0.039736
0.033185	0.164761	0.049876
0.039838	0.051844	0.049622
0.038633	0.074925	0.027261
0.068703	0.045299	
0.032236	0.298709	
0.044569	0.056633	
0.047245		
0.057744		
0.047201		
0.034503		
0.020314		

Fig 3I		
FA/FA	$PM_{2.5}$/FA	FA/$PM_{2.5}$
3.353387	3.434191	3.440787
3.965266	3.247858	3.830201
3.808388	2.848579	3.715243
3.841319	3.730142	3.699537
3.705446	3.512705	4.197697
3.370863	3.829039	
3.969644	2.358996	
3.751007	3.620493	
3.767351		
3.601518		
3.689047		
3.836374		
4.368595		

Females_Data

Fig 3F		
FA/FA	$PM_{2.5}$/FA	FA/$PM_{2.5}$
2.6513	1.137	2.0527
1.7235	2.8526	2.1189
1.8221	2.0858	1.2342
1.1694	2.7183	1.7235
1.8549	3.3255	1.5598
1.7564	1.9867	1.3317
1.7235	1.5598	1.4293
1.137	3.2077	
1.137	3.0547	
1.2667	1.6908	
1.137	1.5598	
1.6253		
1.2018		
2.2846		
1.8549		
1.6908		
1.8878		
2.4509		

Fig 4D	
c_Bacteroidia	4.92252
c_Epsilonproteobacteria	4.01313
c_Vampirovibrio_c	3.87471
c_Verrucomicrobiae	-5.01456
f_Akkermansiaceae	-5.01456
f_Helicobacteraceae	4.01313
f_Muribaculaceae	4.69606
g_Akkermansia	-5.01456
g_Alloprevotella	-3.31186
g_Eubacterium_g23	-4.35909
g_Helicobacter	4.01313
g_Olsenella	-3.27608
g_Oscillibacter	3.59096
g_Ruminococcus	3.47432
o_Bacteroidales	4.92252
o_Campylobacterales	4.01313
o_Verrucomicrobiales	-5.01456
p_Bacteroidetes	4.92252
p_Cyanobacteria	3.87471
p_Verrucomicrobia	-5.01456

Fig 4F	
c_Verrucomicrobiae	-5.02634
f_Akkermansiaceae	-5.02634
f_Odoribacteraceae	-4.08666
g_Akkermansia	-5.02634
g_Eubacterium_g23	-4.34547
g_Odoribacter	-4.08666
o_Verrucomicrobiales	-5.02634
p_Verrucomicrobia	-5.02634
c_Bacteroidia	4.97854
c_Erysipelotrichi	3.58341
f_Erysipelotrichaceae	3.58341
f_Rikenellaceae	4.36552
g_Alistipes	4.41899
g_Clostridium_g24	3.17999
g_Muribaculum	4.35322
g_Ruminococcus	3.35398
g_Turicibacter	3.48938
o_Bacteroidales	4.97854
o_Erysipelotrichales	3.58341
p_Bacteroidetes	4.97854

Fig 4E	
c_Erysipelotrichi	-3.60306
f_Rikenellaceae	-4.32843
f_Erysipelotrichaceae	-3.60306
g_Turicibacter	-3.59967
g_Alistipes	-4.32843
g_Clostridium_g24	-2.70472
g_Muribaculum	-4.20484
o_Erysipelotrichales	-3.60306
f_Odoribacteraceae	4.13416
f_Mogibacterium_f	2.66558
g_Odoribacter	4.13416

Females_Fig 1H 2B 2D

Fig 1H

FA/FA					PM$_{2.5}$/FA				
3.29	3.09	3.50	2.78	3.55	4.45	4.63	4.91		
3.34	2.90	3.42	2.70	3.30	3.04	4.58	3.56		
3.39	3.25	3.51	2.71	3.59	3.05	4.59	3.57		
3.33	3.08	3.51	2.78	3.55	3.02	4.58	3.56		
3.29	3.11	3.67	2.92	3.76	3.12	3.89	3.27		
3.30	3.15	3.32	2.99	3.57	3.21	4.52	3.80		
3.18	3.34	3.65	3.04	3.78	3.58	3.14	3.86		

FA/PM$_{2.5}$				
4.26	3.86	3.47	3.56	3.87
4.24	3.84	3.45	3.54	3.84
4.15	3.85	3.45	3.55	3.85
4.09	3.84	3.46	3.64	3.85
5.10	4.01	3.15	3.67	3.94
4.21	3.78	3.67	3.46	3.74
3.95	3.80	3.50	3.40	3.88

Fig 2B

	FA/FA						PM$_{2.5}$/FA		
AgRP	0.189202	0.169811	0.180616	0.146148	0.273763	0.417255	0.29331	0.367929	0.657471
NPY	0.387697	0.41151	0.427946	1.301793	0.330106	0.748980	0.704416	0.709562	1.035983
POMC	0.398044	0.393518	0.391342	0.128203	0.309498	0.416966	0.34329	0.369207	0.499134

	FA/PM$_{2.5}$		
AgRP	0.430027	0.255342	0.333094
NPY	0.664343	0.500000	0.331252
POMC	0.384352	0.370103	0.195874

Females_Fig 1H 2B 2D

	Fig 2D									
	FA/FA									
TLR4	0.01563	0.010492	0.015522	0.021315	0.013635	0.015893				
ikke	0.001046	0.000763	0.001822	0.000988	0.002237	0.001191				
TNFα	0.00131	0.01041	0.00226	0.00137	0.02525	0.01805	0.00776	0.01278	0.02838	0.02562
	$PM_{2.5}$/FA									
TLR4	0.04616	0.020172	0.04492	0.03782	0.03188	0.02273	0.017	0.01296		
ikke	0.00188	0.001034	0.001934	0.001063	0.002558					
TNFα		0.06393			0.00351	0.01565	0.0559			
	FA/$PM_{2.5}$									
TLR4	0.02462	0.020725	0.026169	0.01933	0.01568	0.02991	0.01839			
ikke	0.000933	0.000335	0.000562	0.001653						
TNFα			0.00464	0.00611	0.04207	0.01189	0.01761			
